# Anxiety and depression relationship with coronary slow flow

**DOI:** 10.1371/journal.pone.0221918

**Published:** 2019-09-05

**Authors:** Ahmed A. Elamragy, Amr A. Abdelhalim, Mohamed E. Arafa, Yasser M. Baghdady

**Affiliations:** 1 Cardiology Department, Cairo University, Cairo, Egypt; 2 Cardiology Department, National Heart Institute, Giza, Egypt; 3 Psychiatry Department, Cairo University, Cairo, Egypt; University of Bologna, ITALY

## Abstract

**Background:**

Psychiatric disorders (depression / anxiety) are linked to coronary artery disease (CAD). Coronary slow flow (CSF) is a relatively common form of CAD with the same underlying mechanisms that are attributed to many anatomic and pathophysiologic factors. However, the relationship between psychiatric disorders and CSF is less well-established; and this is the aim of this study.

**Methods:**

This cross-sectional observational study was conducted on the first 50 consecutive patients diagnosed with CSF by elective coronary angiography (CAG). They were compared with another 50 consecutive patients showing normal coronaries by CAG. Beck Anxiety Inventory and Beck Depression Inventory were used for assessment. CSF was diagnosed by coronary angiography “Thrombolysis In Myocardial Infarction” frame count. Lipid profile was obtained for all patients.

**Results:**

Traditional risk factors (male gender, smoking, total cholesterol, low-density lipoproteins and triglycerides) were higher in the CSF group. Depression and anxiety scores were also higher in the CSF group. On multivariate analysis, male gender, depression and high triglycerides were the only significant independent predictors of CSF. A significant correlation existed between CSF and both anxiety and depression scores. Both scores were also significantly higher in multivessel vs single vessel affection.

**Conclusion:**

Psychiatric depression, male gender and high triglycerides are highly associated with CSF in patients undergoing elective CAG. There is a significant correlation between CSF severity and the severity of both anxiety and depression. Further studies are warranted to explore the impact of psychological intervention on CSF and its long-term outcome.

## Introduction

The incidence of depression is 30%–40% of the general population [1] and 20%–40% of coronary artery disease (CAD) patients; and is associated with increased major adverse cardiovascular events (MACE).[2] As a risk factor, both psychological stress and depression are as important as smoking and more important than diabetes mellitus (DM); accounting for 32.5% of attributable risk.[3] The proposed pathophysiological mechanisms include: increased sympathetic tone, increased cortisol and catecholamines, endothelial dysfunction, release of inflammatory mediators, platelet activation, decreased heart rate variability, accelerated atherogenesis, and poor platelet adherence.[4] Every 5-point increase in Beck Depression Inventory (BDI) score is associated with a 25–30% increase in the risk of definite CAD or abnormal coronary angiography (CAG) findings.[5]

Coronary slow flow (CSF) is defined as the slow progression of angiographic contrast in the coronary arteries in the absence of stenosis in the epicardial vessels.[6] This may be seen in a single coronary artery or more. [7] It is seen in 1–7% of patients undergoing CAG[8] which is the only method for diagnosis.[9] It affects young male smokers most commonly[10] and is linked to both CAD (myocardial ischemia symptoms, life-threatening arrhythmias, recurrent acute coronary syndromes and sudden cardiac death) and psychological manifestations (anxiety, depression and psychological distress).[11–13] The pathogenesis of CSF involves mechanisms similar to those linked to anxiety/depression (inflammation, microvascular abnormalities, endothelial dysfunction and anatomical factors of epicardial arteries).[10] Moreover, some studies indicate that patients with CSF have increased psychological distress compared with patients having coronary normal flow (CNF). [14] However, to our knowledge, very few data are available on the relationship between CSF and psychological disorders; and that is the aim of this study.

## Material and methods

### Study population

This is a cross-sectional observational study conducted between November 2016 and April 2017. We examined 50 consecutive patients who had undergone CAG (due to objective evidence of ischemia: positive exercise stress test or radionuclide study) that revealed CSF and compared them to a control group comprised of 50 consecutive patients with normal CAG performed during the same time interval. Exclusion criteria included: known CAD or acute coronary syndrome; severe valvular heart disease; LV dysfunction defined as ejection fraction <50%; rhythm other than sinus; end-stage renal or hepatic disease; significant chronic obstructive lung disease; treatment for any type of psychiatric disorders; coronary artery stenosis >20%, coronary artery ectasia or any type of coronary abnormality; and uncooperative patients.

### Clinical assessment

All patients were assessed for the presence of CAD risk factors as well as body mass index (BMI) which was calculated by dividing the body weight (in kilograms) over the square of height (in meters). A 12-hour fasting blood sample was withdrawn to measure total cholesterol, triglycerides (TG), high-density lipoprotein (HDL), and low-density lipoprotein (LDL). Electrocardiogram was done to exclude any non-sinus rhythm and echocardiography was done to exclude any valvular heart disease or LV dysfunction. Hypertension was defined as the use of antihypertensive drugs or initial blood pressure over 140/90 mmHg. DM was defined as the use of antidiabetic drugs or fasting blood glucose level > 126 mg/dl. Smoking status was documented: current tobacco use, or ex-smoker (if stopped > 2 years). Obesity was defined as BMI > 30 kg/m^2^. Dyslipidemia was defined as: LDL > 115 mg/dL, and/or TG >150 mg/dL and/or HDL ≤ 40 mg/dL (in males) and ≤ 48 mg/dL (in females).[15]

### Coronary angiography

Selective CAG was performed to all patients through the femoral or radial approach using the Judkins system for cannulation of the right & left coronary arteries. Multiple views were obtained with visualization of the left anterior descending (LAD) and left circumflex (LCx) coronary arteries in at least 4 projections and the right coronary artery (RCA) in at least 2 projections. Angiograms were interpreted by two experienced cardiologists. Normal CAG was defined as the absence of any obstructive/stenotic lesions in any major epicardial arteries. Thrombolysis in myocardial infarction (TIMI) frame count (TFC) was calculated for every major coronary artery to determine CSF as described by Gibson et al.[9] The filming speed was at 15 frames/second, while the standard speed is 30 frames/second, so the frame count was corrected by multiplying by 2. The LAD TFC was corrected by division by 1.7 because this artery is generally longer than the others. Total TFC was calculated as the sum of TFC in the 3 major epicardial vessels (LAD, LCx and RCA). Mean TFC was calculated by dividing the total TFC by 3. Definition of CSF: mean TFC > 27.[9]

### Psychiatric assessment

Psychiatric interviews were performed blinded to the CAG results, to assess the severity of anxiety and depression using the following scales:

#### Beck anxiety inventory (BAI) [16]

This scale measures anxiety in subjects ≥17 years of age and takes 5–10 minutes to complete its 21 self-reported items.[16] Item scores are summed and higher scores indicate higher anxiety levels. Score of 0–21: low anxiety; 22–35: moderate anxiety and ≥ 36: potentially concerning anxiety levels.

#### Beck Depression Inventory (BDI) [17]

This 21-item scale assesses depression severity.[17] Item scores were summed. Score: 1–12: normal, 13–20: mild depression, 21–30: moderate depression and >30: severe depression.

### Ethics

All the procedures were in accordance with the standards of the Institute’s ethical committee and with the Helsinki Declaration of 1975, as revised in 2000. Verbal consent was obtained from the patients during their psychiatric tests by the investigator who documented it in the patients’ files. It was also witnessed by the psychiatric consultant who supervised the BAI and BDI questionnaires. The study design and the verbal consent were approved by the Department of Cardiology—Faculty of Medicine—Cairo University) ethics committee. Cairo University review board approved the whole study.

### Statistical methods

The collected data were coded, tabulated, and statistically analyzed using Statistical Package for Social Sciences software version 23. Data were normally distributed. Continuous data were presented as mean ± standard deviation (SD) and minimum & maximum of the range, while categorical data were presented as number (percentage). Comparison between continuous data in the 2 groups was done using independent sample t-test and Chi-square test /Fisher Exact test for categorical data. Correlation between continuous variables was done using Pearson’s correlation coefficient (r). Significance was considered at *p* < 0.05. Significantly associated variables with CSF entered a multivariate logistic regression analysis to obtain the most significant independent predictors of CSF with the relevant odds ratio (OR) and 95% confidence interval (CI).

## Results

### Baseline characteristics

One hundred patients were enrolled in this study (50 in each group). Demographic data, risk factors and CAG results are summarized in Table 1. Male gender, smoking, total cholesterol, LDL and TG were higher in the CSF group. CSF patients had significantly higher BAI and BDI scores; most of the patients had moderate–severe anxiety levels.

**Table 1:** Demographic data, risk factors, coronary angiography results and psychiatric test results in study groups.

	CNF (n = 50)	CSF (n = 50)	*p* value
**Atherosclerotic risk factors**			
Male Gender, n (%)	14 (28)	29 (58)	**0.002**^a^
Age (years)	52.7 ± 10.3	51.7 ± 10.4	0.6
DM, n (%)	14 (28)	14 (28)	1.0
Hypertension, n (%)	28 (56)	30 (60)	0.685
Smoking, n (%)	6 (12)	21 (42)	**0.001**^a^
Positive family history, n (%)	16 (32)	16 (32)	1.0
BMI	30.8 ± 5.1	33.1 ± 6.3	0.05
Total Cholesterol	148.2 ± 44.0	182.6 ± 45.4	**< 0.001**^a^
HDL	38.4 ± 16.5	38.1 ± 8.4	0.92
LDL	89.3 ± 39.3	111.7 ± 32.0	**0.002**^a^
Triglycerides	102.6±41	149±84.7	**< 0.001**^a^
**Number of vessels with CSF**			
Single vessel, n (%)		24 (48%)	
Multivessel, n (%)		26 (52%)	
**TFC**			
-LAD	16.4 ± 4.6	25 ± 9.6	**< 0.001**^a^
-LCX	21.0 ± 3.6	34.5 ± 9.9	**< 0.001**^a^
-RCA	17.9 ± 3.9	29.6 ± 12.5	**< 0.001**^a^
-Mean TFC	18.5 ± 3.0	29.7 ± 8.1	**< 0.001**^a^
-Total TFC	55.5 ± 9.1	89.1 ± 24.3	**< 0.001**^a^
**BAI**	18.3 ± 8.1	25 ± 8.6	**< 0.00**^a^
Low anxiety: 0–21, n (%)	36 (72%)	21 (42%)	**0.009**^a^
Moderate anxiety: 22–35, n(%)	12 (24%)	23 (46%)	
Severe anxiety: >35, n (%)	2 (4%)	6 (12%)	
**BDI**	11.1 ± 4.8	17.9 ± 8.6	**< 0.001**^a^
0–12 (Normal)	30 (60)	13 (26)	**<0.001**^a^
Mild depression: 13–20, n(%)	17 (34)	19 (38)	
Moderate depression: 21–30, n (%)	3 (6)	16 (32)	
Severe depression: ≥ 35, n (%)	0 (0)	2 (4)	

CNF: coronary normal flow, CSF: coronary slow flow, DM: Diabetes mellitus, BMI: body mass index LAD: left anterior descending artery, LCX: left circumflex artery, RCA: Right coronary artery, HDL: high-density lipoprotein, LDL: low-density lipoprotein, TFC: “thrombolysis in myocardial infarction” frame count. BAI: Beck anxiety inventory, BDI: Beck depression inventory. Data is presented as mean± standard deviation for continuous variables and numbers (percentage) for categorical ones.

^**a**^ Significant *p* value (<0.05)

### TFC correlation analysis

The mean TFC had significant strong correlation with both BAI and BDI scores and significant but weak correlation with TG and BMI. However, there was no correlation with the other atherosclerotic risk factors. (Table 2).

**Table 2:** Correlation between the TFC and (BAI, BDI).

TFC	r	*p* value
BAI	0.621	**< 0.001**^a^
BDI	0.719	**< 0.001**^a^
BMI	0.256	**0.01**^a^
Age	0.013	0.9
Total Cholesterol	0.167	0.098
HDL	-0.088	0.385
LDL	0.111	0.274
TG	0.227	**0.023**^a^

TFC: “Thrombolysis in myocardial infarction” frame count, r: Pearson’s correlation coefficient, BAI: Beck anxiety inventory, BDI: Beck depression inventory, BMI: Body mass index, TG: Triglycerides.

^**a**^ Significant *p* value (<0.05)

### Multivariate analysis

A multivariate stepwise regression model using significant univariate variables (gender, smoking, LDL, TG, BMI, BDI and BMI) revealed that the only significant independent determinants of CSF were male gender, BDI and TG. (Table 3).

**Table 3:** Independent predictors of coronary slow flow.

Variable	OR	95% CI	*p* value
Male gender	3.5	1.3–9.7	0.016^a^
BDI	1.2	1.1–1.3	0.001^a^
TG	1.01	1.0–1.02	0.0037^a^

OR: odds ratio, CI: Confidence interval, BDI: Beck depression inventory, TG: triglycerides.

^**a**^ Significant *p* value (<0.05)

### Subgroup analysis

#### Gender stratification

Male gender was an independent determinant of CSF, but it was more prevalent in the CSF group (58% vs 28%) and had non-significantly higher BAI and BDI scores than females (22.3 ± 10.5 vs 21.1 ± 7.8, p: 0.53 and 15.9 ± 9.1 vs 13.4 ± 6.4, p: 0.11 respectively). Despite gender stratification to eliminate its effect, BAI and BDI were still significantly higher in the CSF group. (Table 4).

**Table 4:** Gender stratification.

	Female		p-value	Male		*p* value
	CNF (n = 36)	CSF (n = 21)		CNF (n = 14)	CSF (n = 29)	
**BAI**	19.17 ± 7.0	24.52 ± 8.0	**0.015**^a^	15.93 ± 10.4	25.34 ± 9.2	**0.008**^a^
**BDI**	11.78 ± 5.0	16.19 ± 7.7	**0.019**^a^	9.21 ± 3.8	19.17 ± 9.2	**<0.001**^a^

CNF: coronary normal flow, CSF: coronary slow flow, BAI: Beck anxiety inventory, BDI: Beck depression inventory.

^**a**^ Significant *p* value (<0.05)

#### Number of vessels with CSF

Patients with multiple-vessel CSF had significantly higher BAI & BDI scores than those with single-vessel CSF. (Table 5).

**Table 5:** BAI & BDI scores in single versus multivessel coronary slow flow.

	CSF in a single vessel (n = 24)	CSF in multiple vessels (n = 26)	*p* value
**BAI**	21 ± 5.4	28.7 ± 9.5	**0.001**^a^
**BDI**	14 ± 4.8	21.5 ± 9.8	**0.001**^a^

CSF: coronary slow flow, BAI: Beck anxiety inventory, BDI: Beck depression inventory.

^**a**^ Significant *p* value (<0.05).

## Discussion

The present study shows that CSF is significantly and independently associated with male gender and increased TG levels. Male gender is the most important independent predictor of CSF (OR:3.5, 95% CI: 1.27–9.7, p = 0.016). Other significant but not independent associations included: smoking, LDL and a trend with obesity. These are traditional CAD risk factors which supports the theory of subclinical atherosclerosis role in CSF pathogenesis (involving increased sympathetic activity, inflammatory response, endothelial dysfunction, abnormal platelet activation and release of its products).[4] However, previous studies examining this association showed conflicting results: male gender and high TG levels were significant independent predictors of CSF in some studies,[18,19] while others found no relationship between gender and CSF.[20] As for high TG, the present study is consistent with previous one by Dai YX et al, who reported that patients with high levels of TG (in addition to high fasting blood glucose and low levels of HDL) were more likely to have CSF. [21] In contrast, DM–an important CAD risk factor associated with endothelial dysfunction- was not associated with CSF. Previous studies showed similar results[22–24]. This may be explained by the similar rate of DM in both groups, so it could not enter in the multivariate regression analysis and act as a predictor of CSF.

In the present study, CSF is also significantly associated with depression and anxiety, irrespective of the gender. Depression is the second most important independent predictor for CSF: every unit increase in the BDI score is associated with 1.2 times increase in the CSF probability (*p* = 0.001), while anxiety has also a significant but dependent association with CSF. These findings are concordant with the INTERHEART study[3]; the largest trial to study the relationship between psychiatric disorders and heart disease. It showed that stress and depression were important risk factors for CAD; accounting for 32.5% of the attributable risk. Similarly, Vural et al.[5] estimated that every 1-point increase in BDI score was associated with a 5–6% increase in the risk of definitive CAD or abnormal CAG findings. Noticeably, the present study has more pronounced results: every point increase in BDI is associated with 20% probability increase in CSF and CSF severity as well as the number of involved vessels is positively correlated with the severity of depression and anxiety. This is because the study is restricted to patients with CSF only (more specific patient cohort). Previous data about the relationship between coronary circulation physiology and depression is conflicting. Yang et al. demonstrated that depression was not associated with coronary endothelial dysfunction[25], while Sherwood et al. reported that vascular endothelial dysfunction was present in CAD patients with symptoms of depression.[26]

Shiozaki et al. showed that depression following myocardial infarction was significantly associated with subsequent MACE during follow up.[27] In the present study, this is not addressed because the aim is to establish an association between depression and CSF and not to study long-term consequences of depression.

The relationship between anxiety and CAD is conflicting. Anxiety was associated with an increased risk of sudden cardiac death and fatal CAD in a previous study.[28–30] Patients with baseline generalized anxiety disorder (GAD) had more subsequent MACE than those without GAD.[31] In contrast, there was no association between anxiety and CAD in another study; but the highest BAI scores were in patients with CSF and/or non-obstructive CAD.[5] In the present study, CSF patients have significantly higher BAI scores and a strong correlation exists between BAI score and CSF severity. Moderate and severe anxiety levels are more common in the CSF group (58% vs 28%) and anxiety severity is significantly correlated with CSF severity and the number of affected vessels. Durmaz et al. showed similar results using the State-Trait Anxiety Inventory (STAI) and showed similar results.[12] BAI score is as reliable as STAI with the advantage of stratifying patients into mild, moderate and severe anxiety.

Depression and anxiety are more prevalent and their scores are higher in women than men[17,32–36]. In contrast, the present study showed similar scores in both genders. Another study examined gender differences in depression (using the BDI score) and revealed that the differences may be possibly related to the instruments used for measurement[37]. Another explanation is that the previous studies included a different cohort: some studied patients with major depression illness while the National Comorbidity Survey[32–34] examined normal population with a wide age range including adolescents. The present study included a different cohort: patients with CAD with no history of psychiatric illness and a mean age: 52 ± 10.3 years.

The relationship between anxiety/depression and body weight is also controversial in the literature. Previous studies showed that obese and overweight people were more likely to have anxiety and depression than those with normal weight.[38] CNF patients were overweight, whereas patients with CSF were obese (as measured by BMI)[14]. In the present study, CSF severity is weakly correlated with BMI. There is also a weak correlation between BMI and both BDI and BAI scores, but investigating this relationship is not the primary aim of this study. Therefore, it may not be suitable to draw conclusions in this issue.

### Study limitations

The present study is a cross-sectional observational study and thus cannot conclude a definite causal relationship between CSF and depression or anxiety disorder. There is no follow up. Thus, we cannot correlate the study parameters with long-term outcomes. The anxiety/depression scales are based on patients’ subjective responses; which may not be valid if patients do not provide reliable answers. Integrating BAI and BDI scores with clinical evaluation would have evaded the subjective nature of these scores providing better assessment of the psychiatric status. Antidepressants and anti-anxiety medications are not accounted for in the analysis of results. However, none of these medications is known to cause CSF. The use of anti-ischemic drugs like beta-blockers, vasodilators and others were also not reported; this might have some influence on CSF severity. Finally, some non-medical factors that may affect the psychological state of patients are not considered, e.g. socio-economic status, education state, etc.

### Future recommendations

Patients diagnosed with CSF need to have their psychological wellbeing properly assessed; to develop care plans to meet their psychological needs, and to refer to mental health care departments if needed to avoid any physical or psychological complications. We need further studies to assess the impact of psychological intervention on CSF and clinical outcome.

## Conclusion

Psychiatric depression, male gender and high TGs are highly associated with CSF in patients with no obstructive CAD on elective CAG. There is a significant correlation between CSF severity and the severity of both anxiety and depression. Further studies are warranted to explore the impact of psychological intervention on CSF.
