# Supplementary material for: Anxiety and depression relationship with coronary slow flow
Source: PLoS One. 2019 Sep 5;14(9):e0221918. doi: 10.1371/journal.pone.0221918 (PMC6728014; doi:10.1371/journal.pone.0221918)
Supplement: Peer review history

# Peer review: Decision Letter 0

**DOI:** 10.1371/journal.pone.0221918.r001

**Authors:** Carmine Pizzi

28 Jun 2019

PONE-D-19-14921

Anxiety and Depression Relationship with Coronary Slow Flow

PLOS ONE

Dear Dr. Elamragy,

Thank you for submitting your manuscript to PLOS ONE. After careful consideration, we feel that it has merit but does not fully meet PLOS ONE’s publication criteria as it currently stands. Therefore, we invite you to submit a revised version of the manuscript that addresses the points raised during the review process.

We would appreciate receiving your revised manuscript by Aug 12 2019 11:59PM. When you are ready to submit your revision, log on to https://www.editorialmanager.com/pone/ and select the 'Submissions Needing Revision' folder to locate your manuscript file.

If you would like to make changes to your financial disclosure, please include your updated statement in your cover letter.

To enhance the reproducibility of your results, we recommend that if applicable you deposit your laboratory protocols in protocols.io, where a protocol can be assigned its own identifier (DOI) such that it can be cited independently in the future. For instructions see: http://journals.plos.org/plosone/s/submission-guidelines#loc-laboratory-protocols

Please include the following items when submitting your revised manuscript:

- A rebuttal letter that responds to each point raised by the academic editor and reviewer(s). This letter should be uploaded as separate file and labeled 'Response to Reviewers'.
- A marked-up copy of your manuscript that highlights changes made to the original version. This file should be uploaded as separate file and labeled 'Revised Manuscript with Track Changes'.
- An unmarked version of your revised paper without tracked changes. This file should be uploaded as separate file and labeled 'Manuscript'.

Please note while forming your response, if your article is accepted, you may have the opportunity to make the peer review history publicly available. The record will include editor decision letters (with reviews) and your responses to reviewer comments. If eligible, we will contact you to opt in or out.

We look forward to receiving your revised manuscript.

Kind regards,

Carmine Pizzi

Academic Editor

PLOS ONE

Journal Requirements:

1. When submitting your revision, we need you to address these additional requirements.

Please ensure that your manuscript meets PLOS ONE's style requirements, including those for file naming. The PLOS ONE style templates can be found at

http://www.journals.plos.org/plosone/s/file?id=wjVg/PLOSOne_formatting_sample_main_body.pdf and http://www.journals.plos.org/plosone/s/file?id=ba62/PLOSOne_formatting_sample_title_authors_affiliations.pdf

2. Thank you for including your ethics statement; "All the procedures were in accordance with the standards of the Institute’s ethical committee and with the Helsinki Declaration of 1975, as revised in 2000. Oral consent was taken from the subjects participated in the psychiatric questionnaires."

We note that you include the full name of your Institutional Review Board on the online submission form; "Cairo University Ethics committee approved the study.

Oral consent was obtained."

Please amend your current ethics statement to include the full name of the ethics committee/institutional review board(s) that approved your specific study and also ensure that you have specified the committee approved the study.

Also, please provide additional details regarding participant consent and ensure that you have specified how the verbal or oral consent was documented and witnessed. If the verbal consent was approved by the ethics committee, please include this information.

Once you have amended this/these statement(s) in the Methods section of the manuscript, please add the same text to the “Ethics Statement” field of the submission form (via “Edit Submission”).

For additional information about PLOS ONE ethical requirements for human subjects research, please refer to http://journals.plos.org/plosone/s/submission-guidelines#loc-human-subjects-research.

Reviewer #1: In this study, the authors aimed to assess the possible relation of psychiatric disorders, such as depression and anxiety status, with the coronary flow pattern in patients with angiographically normal coronary arteries. The TIMI frame count (TFC) has been used for definition of coronary slow flow (CSF) phenomenon and psychiatric interviews were performed to assess severity of anxiety and depression using the BAI and BDI scales. The results show that patients with CSF had significantly higher anxiety and depression scores, irrespective of gender. The authors also found a strong correlation between the BAI score and CSF severity.

We thank the authors for this interesting study. The pathophysiologic hypothesis is intriguing and the study design is fitting. However, this reviewer has several concerns:

Major comments

- The authors should give detailed information about the clinical findings of CSF patients and control patients. In particular, the symptomatic burden, including duration of the angina pattern, as well as frequency and severity of angina attacks should be reported and compared.

- The use of beta-blockers, vasodilators or other anti-ischemic drugs might have affected the detection of CSF. The authors should at least comment this point as a limitation of the study.

- If possible, the authors should report the educational and socioeconomic status of patients of the 2 groups, as possible factors influencing depression or anxiety status.

Minor comments

- The analysis of this study doesn’t account for the use of psychiatric drugs or other medication. Although none of the antidepressant drugs is known to cause CSF, as the authors say in the text, the answers of patients to the interviews might have been influenced by their use.

- Have the authors tried to exclude patients with possible “secondary form” of CSF (i.e. coronary artery ectasia, coronary artery spasm, pulmonary arterial hypertension, etc...)?

- The depression and anxiety rating scales (BAI and BDI), used to assess the severity of the psychiatric disorder, are two self-report scales. Thus, they depend on patients’ reliability, time and context of interview administration.

Reviewer #2: The study is focused on a large-scale problem that is the increasing prevalence of patients with both coronary artery disease (CAD) and depression or anxiety, that, to date, are probably not well diagnosed and treated for the underlying psychological conditions despite growing evidences that these conditions play an important role in provoking and modifying the prognosis in cardiovascular diseases. The study, taking into account the intrinsic limitations of a cross sectional study, shows significant and independent association between depression and coronary slow flow (CSF) as well as with male gender or triglyceride serum levels (association already demonstrated by other authors) and significant but dependent association with anxiety and other classical cardiovascular risk factors. The paper, sustaining the role of psychological conditions together with classical cardiovascular risk factors in determining CSF, wants to stress the importance of an accurate global (physical and psychological) assessment of the patient. An interesting point of the paper is the exclusion of patients with a coronary artery stenosis > 20%, in fact even mild-to-moderate stenosis could affect CSF and could have represented a selection bias.

However, this manuscript had some issues that deserve clarifications.

Major comments

1. The study population is composed by consecutive patients who underwent coronary angiography due to ‘objective evidence of ischemia’. What do the Authors mean with that sentence? It is necessary to provide a clear definition of ischemia and a standardized diagnosis.

2. Diabetes, one of the most important cardiovascular risk factors and strongly related to endothelial dysfunction, seems not to be associated with CSF. The authors should be discussing this point

3. Another point is the definition of the ex-smoker status. The Authors have provided an arbitrary definition of former smoker (only the patients who have stopped > 2 years), not shared by the principal National Health Systems. This gross classification of patients probably leads to some bias in defining the relationship between smoking and CSF because the toxic substances contained in cigarettes act in a short/medium period of time in causing endothelial dysfunction and inflammation.

4. It is well established that depression and anxiety are twice as high in females than in males, in this study the mean BDI and BAI scores are lower in women. Maybe these scores should be integrated with a clinical evaluation of symptoms by a psychiatrist to avoid the subjective influence of the patients and on the physician in interpreting the answers. It would also be useful to provide the complete data of the prevalence and of the degree of anxiety and depression adjusted for gender.

5. One major problem of this work is that it does not evaluate the role of psychoactive drugs. For example, it is not well established if the antidepressants play a role in unmasking endothelial dysfunction or platelet adhesion and it is still a matter of debate the role of these drugs in improving symptoms and prognosis in patients with cardiovascular diseases. It would be interesting to have some data regarding this issue in the study population.

Minor comments

1. Please check the references. For example, references 10-15 refer to articles that do not match with the text.

2. Try to make the ‘Result Section’ more uniform. There are many tables that can be merged into one single table. This solution will help in improving paper’s structure: more logical and fluid to read and understand.

# Author comment: Author response to Decision Letter 0

**DOI:** 10.1371/journal.pone.0221918.r002

3 Aug 2019

Reviewer #1: 

Major comments

- The authors should give detailed information about the clinical findings of CSF patients and control patients. In particular, the symptomatic burden, including duration of the angina pattern, as well as frequency and severity of angina attacks should be reported and compared.

Response: 

I added a definition to the methodology explaining what we meant by objective evidence of ischemia. Duration and frequency of angina information (for all patients) are not available in the database of this study, only the results of the non-invasive tests for coronary artery disease.

- The use of beta-blockers, vasodilators or other anti-ischemic drugs might have affected the detection of CSF. The authors should at least comment this point as a limitation of the study.

Response:

I added this comment to the limitation.

- If possible, the authors should report the educational and socioeconomic status of patients of the 2 groups, as possible factors influencing depression or anxiety status.

Response:

Unfortunately, data on educational and socioeconomic status of patients are not available in our database, and this is mentioned in the limitations section. However, our institution mainly serves patients of low-intermediate socio-economic class.

Minor comments

- The analysis of this study doesn’t account for the use of psychiatric drugs or other medication. Although none of the antidepressant drugs is known to cause CSF, as the authors say in the text, the answers of patients to the interviews might have been influenced by their use.

Response:

One of the exclusion criteria is the presence of psychiatric illness or the regular use of psychiatric drugs. So, this issue is unlikely to cause any bias. However, we mentioned it in the limitation section.

- Have the authors tried to exclude patients with possible “secondary form” of CSF (i.e. coronary artery ectasia, coronary artery spasm, pulmonary arterial hypertension, etc...)?

Response:

Yes, we excluded all patients with ectasia or spasm or known gross heart or vascular disease.

- The depression and anxiety rating scales (BAI and BDI), used to assess the severity of the psychiatric disorder, are two self-report scales. Thus, they depend on patients’ reliability, time and context of interview administration.

Response: 

This is an absolutely valid point, which we mentioned in the limitation section. For a better assessment of psychiatric status, this should have been correlated with clinical symptoms.

Reviewer #2: 

Major comments

1. The study population is composed by consecutive patients who underwent coronary angiography due to ‘objective evidence of ischemia’. What do the Authors mean with that sentence? It is necessary to provide a clear definition of ischemia and a standardized diagnosis.

Response: 

A statement was added to the paragraph defining the objective evidence of ischemia as positive exercise stress test or radionuclide study. 

2. Diabetes, one of the most important cardiovascular risk factors and strongly related to endothelial dysfunction, seems not to be associated with CSF. The authors should be discussing this point

Response:

I added this point to the discussion.

3. Another point is the definition of the ex-smoker status. The Authors have provided an arbitrary definition of former smoker (only the patients who have stopped > 2 years), not shared by the principal National Health Systems. This gross classification of patients probably leads to some bias in defining the relationship between smoking and CSF because the toxic substances contained in cigarettes act in a short/medium period of time in causing endothelial dysfunction and inflammation.

Response:

Some studies demonstrated that 1-year of smoking cessation was associated with improved endothelial function (Johnson, JACC 2010 May 4;55(18):1988-95). Our database defines former smoking as those who stopped >2 years. We found that this was not a major issue (based on the above-mentioned study); so during patient enrollment, we opted not to change the current definition in our institution’s database. However, this is a valid point to be seriously considered before carrying on future research.

4. It is well established that depression and anxiety are twice as high in females than in males, in this study the mean BDI and BAI scores are lower in women. Maybe these scores should be integrated with a clinical evaluation of symptoms by a psychiatrist to avoid the subjective influence of the patients and on the physician in interpreting the answers. It would also be useful to provide the complete data of the prevalence and of the degree of anxiety and depression adjusted for gender.

Response: 

I added the total scores of BDI and BAI in males and females in both groups to the results section, the difference was not significant. This was different from the well-established fact referred to by the reviewer. I added this to the discussion section stating that this issue may be related to the different cohort used in the study. I also found another study showing similar data, and it was attributed to the difference in the instruments used in analysis of psychiatric illness. Integrating the scores with the clinical evaluation is a very good idea and would give a better assessment of the psychiatric state. Unfortunately, it was not done because it was not part of the study design and methodology. I added this to the limitations section.

5. One major problem of this work is that it does not evaluate the role of psychoactive drugs. For example, it is not well established if the antidepressants play a role in unmasking endothelial dysfunction or platelet adhesion and it is still a matter of debate the role of these drugs in improving symptoms and prognosis in patients with cardiovascular diseases. It would be interesting to have some data regarding this issue in the study population.

Response: None of the patients were on regular psychoactive drugs. This is indeed a major limitation that I mentioned in the limitation section. However, none of the patients were previously diagnosed with psychiatric illnesses or were on regular long-term psychoactive drugs (it was an exclusion criterion).

Minor comments

1. Please check the references. For example, references 10-15 refer to articles that do not match with the text.

Response:

I rechecked and updated all the references after correction of any error.

2. Try to make the ‘Result Section’ more uniform. There are many tables that can be merged into one single table. This solution will help in improving paper’s structure: more logical and fluid to read and understand.

Response:

I revised the results section and merged some of the tables. I hope it looks better now.

Attachment Submitted filename: Response to Reviewers.docx Click here for additional data file.

# Peer review: Decision Letter 1

**DOI:** 10.1371/journal.pone.0221918.r003

**Authors:** Carmine Pizzi

20 Aug 2019

Anxiety and Depression Relationship with Coronary Slow Flow

PONE-D-19-14921R1

Dear Dr. Elamragy,

We are pleased to inform you that your manuscript has been judged scientifically suitable for publication and will be formally accepted for publication once it complies with all outstanding technical requirements.

Within one week, you will receive an e-mail containing information on the amendments required prior to publication. When all required modifications have been addressed, you will receive a formal acceptance letter and your manuscript will proceed to our production department and be scheduled for publication.

Shortly after the formal acceptance letter is sent, an invoice for payment will follow. To ensure an efficient production and billing process, please log into Editorial Manager at https://www.editorialmanager.com/pone/, click the "Update My Information" link at the top of the page, and update your user information. If you have any billing related questions, please contact our Author Billing department directly at authorbilling@plos.org.

If your institution or institutions have a press office, please notify them about your upcoming paper to enable them to help maximize its impact. If they will be preparing press materials for this manuscript, you must inform our press team as soon as possible and no later than 48 hours after receiving the formal acceptance. Your manuscript will remain under strict press embargo until 2 pm Eastern Time on the date of publication. For more information, please contact onepress@plos.org.

With kind regards,

Carmine Pizzi

Academic Editor

PLOS ONE

# Editor report: Acceptance letter

**DOI:** 10.1371/journal.pone.0221918.r004

**Authors:** Carmine Pizzi

26 Aug 2019

PONE-D-19-14921R1 

Anxiety and Depression Relationship with Coronary Slow Flow 

Dear Dr. Elamragy:

I am pleased to inform you that your manuscript has been deemed suitable for publication in PLOS ONE. Congratulations! Your manuscript is now with our production department. 

If your institution or institutions have a press office, please notify them about your upcoming paper at this point, to enable them to help maximize its impact. If they will be preparing press materials for this manuscript, please inform our press team within the next 48 hours. Your manuscript will remain under strict press embargo until 2 pm Eastern Time on the date of publication. For more information please contact onepress@plos.org.

For any other questions or concerns, please email plosone@plos.org. 

Thank you for submitting your work to PLOS ONE.

With kind regards,

PLOS ONE Editorial Office Staff

on behalf of

Dr. Carmine Pizzi 

Academic Editor

PLOS ONE
